# Editorial: Interplay between plant nutrient uptake and abiotic stress

**DOI:** 10.3389/fpls.2026.1860157

**Published:** 2026-05-08

**Authors:** Yingying Huang, Qiong Liao, Chu Zhong

**Affiliations:** 1 School of Chemical and Environmental Engineering, Hunan Institute of Technology, Hengyang, Hunan, China; 2 Hunan Chemical Vocational Technology College, Zhuzhou, China; 3 Guangxi Key Laboratory of Medicinal Resource Protection and Genetic Improvement, Guangxi Botanical Garden of Medicinal Plants, Nanning, China

**Keywords:** abiotic stress, nitrogen use efficiency, nutrient uptake, nutrient–stress interaction, plant resilience

Efficient nutrient uptake is essential for plant growth, development, and productivity. However, plants are constantly exposed to abiotic stresses such as drought, salinity, temperature extremes, and soil degradation, which disrupt nutrient acquisition, transport, and metabolism. These stresses alter root architecture, impair ion homeostasis, and induce oxidative damage, ultimately limiting crop yield and quality. Importantly, these processes collectively reshape the dynamic interplay between nutrient uptake and plant stress responses, highlighting the need for an integrated understanding of their coordination. This Research Topic brings together ten contributions that collectively advance our understanding of how plants regulate nutrient uptake and utilization under abiotic stress, spanning physiological, molecular, and ecological perspectives. Together, these studies reveal a multi-layered regulatory network integrating nutrient metabolism, transport, and environmental interactions under stress conditions.

## Nutrient metabolism and stress responses

A prominent theme emerging from this Research Topic is the central role of nutrient metabolism in plant adaptation to stress. Several studies highlight the tight coordination between carbon and nitrogen metabolism in maintaining cellular homeostasis under adverse conditions. For instance, enhanced nitrogen assimilation and carbon metabolic reprogramming contribute to improved tolerance to low-temperature stress (Jian et al.). Similarly, optimized nitrogen application can promote plant growth and secondary metabolite accumulation through coordinated regulation of physiological and transcriptional processes (Yuan et al.).

Nitrogen use efficiency (NUE) is further emphasized as a critical determinant of plant performance. Variations in root traits, nutrient uptake capacity, and the balance between carbon assimilation and respiration collectively influence NUE, providing important insights for improving crop productivity under stress conditions (Ye et al.). Collectively, these studies demonstrate that metabolic coordination acts as a central hub linking nutrient availability with stress adaptation, enabling plants to maintain growth under adverse conditions.

## Nutrient transport and ionic homeostasis

Maintaining nutrient transport systems and ionic balance is essential for plant resilience. Several contributions focus on the molecular mechanisms underlying nutrient transport and homeostasis. For example, genome-wide analyses of magnesium transporter families reveal their functional diversification and essential roles in nutrient uptake and intracellular transport (Hao et al.). In a broader context, magnesium has been identified as a key regulator of photosynthesis, enzyme activity, and stress adaptation, highlighting its importance in plant resilience to abiotic stress (Sarraf et al.).

In addition, nutrient interactions play a crucial role in stress mitigation. Evidence from field and experimental studies demonstrates that iron supplementation can alleviate manganese toxicity by restoring nutrient balance and enhancing chlorophyll biosynthesis (Li et al.). Together, these findings emphasize that nutrient transport systems and ionic homeostasis are not isolated processes but are tightly integrated with stress signaling networks, forming a coordinated regulatory system for plant adaptation.

## Soil environment and nutrient cycling

The soil environment represents a critical interface influencing nutrient availability and plant responses to stress. Studies in this Research Topic demonstrate that microbial inoculants can accelerate organic matter decomposition, enhance nutrient release, and improve crop productivity, particularly in saline-alkali soils (Wang et al.). These findings highlight the importance of soil microbial activity in regulating nutrient cycling under challenging conditions.

Conversely, emerging environmental factors such as microplastic contamination are shown to disrupt soil nitrogen dynamics, reduce nitrogen use efficiency, and negatively affect crop productivity (Sarfraz et al.). These results emphasize the need to consider complex soil–plant–environment interactions when addressing nutrient uptake under abiotic stress. Taken together, these studies highlight that soil processes act as a critical bridge linking environmental stressors with plant nutrient acquisition, reinforcing the importance of belowground–aboveground integration.

## Integration of multi-omics and agronomic approaches

Advances in high-throughput technologies have enabled deeper insights into the molecular mechanisms governing nutrient–stress interactions. Integrated transcriptomic and metabolomic analyses reveal how fertilization strategies regulate gene expression and metabolic pathways, leading to improved nutrient accumulation and plant growth (Xu et al.). Such multi-omics approaches provide a systems-level framework for dissecting complex nutrient–stress interactions across multiple biological scales.

In addition, agronomic studies on nutrient management strategies, including nitrogen–potassium coupling, demonstrate their potential to enhance crop yield and soil quality under specific environmental conditions (Zhong et al.). These findings bridge the gap between molecular insights and practical applications, contributing to the development of sustainable agricultural systems.

## Future perspectives

The studies compiled in this Research Topic collectively illustrate the complexity of the interplay between nutrient uptake and abiotic stress. Future research should focus on integrating molecular, physiological, and ecological approaches to better understand these interactions. In particular, combining multi-omics technologies with field-based studies will be essential for translating fundamental discoveries into practical applications.

Moreover, future efforts should prioritize the integration of root–microbiome interactions, high-throughput phenotyping, and machine learning approaches to predict plant performance under stress conditions. Improving nutrient use efficiency and optimizing nutrient management strategies, alongside breeding stress-resilient crop varieties, will be crucial for achieving sustainable agricultural production under changing environmental conditions.

## Conclusion

In summary, this Research Topic provides new insights into how plants coordinate nutrient uptake, transport, and metabolism in response to abiotic stress. By integrating findings across multiple scales, these studies contribute to a more comprehensive understanding of plant resilience and offer promising strategies for enhancing crop productivity and sustainability in the face of global environmental challenges.

